# Genistein Reduces Anxiety-like Behavior During Metestrus–Diestrus Phase Without Changing Estradiol or Progesterone Levels in Wistar Rats

**DOI:** 10.3390/metabo15050311

**Published:** 2025-05-06

**Authors:** Juan Francisco Rodríguez-Landa, Oscar Jerónimo Olmos-Vázquez, Carlos Fabrizio Quiñonez-Bailón, Gabriel Guillén-Ruiz, Ana Karen Limón-Vázquez, Jonathan Cueto-Escobedo, Eduardo Rivadeneyra-Domínguez, Blandina Bernal-Morales

**Affiliations:** 1 Laboratorio de Neurofarmacología, Instituto de Neuroetología, Universidad Veracruzana, Xalapa 91190, Veracruz, Mexico; oscarplsstahp1@gmail.com (O.J.O.-V.); fa_carlos_097@hotmail.com (C.F.Q.-B.); gguillen@uv.mx (G.G.-R.); analimon@uv.mx (A.K.L.-V.); bbernal@uv.mx (B.B.-M.); 2 Facultad de Química Farmacéutica Biológica, Universidad Veracruzana, Xalapa 91000, Veracruz, Mexico; edrivadeneyra@uv.mx; 3 Servicio de Transfusión Sanguínea, Hospital General de Boca del Río, Servicios de Salud Instituto Mexicano del Seguro Social (IMSS-Bienestar), Boca del Río 94290, Veracruz, Mexico; 4 Programa de Investigadoras e Investigadores por México, SECIHTI, Instituto de Neuroetología, Universidad Veracruzana, Xalapa 91190, Veracruz, Mexico; 5 Departamento de Investigación Clínica y Traslacional, Instituto de Ciencias de la Salud, Universidad Veracruzana, Xalapa 91190, Veracruz, Mexico; jcueto@uv.mx

**Keywords:** anxiety, premenstrual syndrome, phytoestrogens, alternative therapy, ovarian cycle, rat

## Abstract

**Background**: Premenstrual syndrome is characterized by emotional changes, including anxiety and depression symptoms, which may be treated with anxiolytic and antidepressant drugs, as well as estrogen therapy. However, steroidal estrogen therapy is contraindicated for patients with a potential risk of developing estrogen-dependent cancers through interactions with estrogen receptor α (ERα). Alternatively, genistein produces estrogenic effects in animals and humans at dietary dosages that act on the nuclear and membrane ERα, estrogen receptor β (ERβ), and the G-protein-coupled estrogen receptor (GPER). These receptors are likely involved in the anxiety symptoms observed in premenstrual disorders. The objective of this study was to evaluate the effects of genistein and 17β-estradiol on anxiety-like behavior and the plasma concentrations of estradiol and progesterone throughout the ovarian cycle of Wistar rats. **Methods**: The effect of the administration of 0.09 mg/kg of genistein or 17β-estradiol was evaluated using the elevated plus maze (EPM) test, locomotor activity test (LAT), and light/dark box (LDB) test, as well as by assessing the plasma concentrations of estradiol and progesterone, while considering the ovarian cycle phases. **Results**: Higher levels of anxiety-like behavior were detected in the metestrus–diestrus phase compared to the proestrus–estrus phase, which was associated with low concentrations of estradiol. Genistein, similarly to 17β-estradiol, significantly reduced anxiety-like behaviors in the EPM and LDB; however, 17β-estradiol, but not genistein, significantly increased the plasma estradiol concentration. No significant changes were found in locomotor activity or the plasma progesterone concentrations due to the treatments. **Conclusions**: These findings suggest that genistein may be useful in the development of alternative therapies to reduce the anxiety associated with low steroid hormone concentrations, which occur in premenstrual syndrome. Genistein could be an alternative to steroidal estrogen therapy to avoid potential side effects due to estradiol or antidepressant treatments, although it still requires medical care.

## 1. Introduction

Anxiety disorders affect a higher percentage of women than men worldwide [1,2]. Multiple factors contribute to these sex differences; however, the factors that stand out include biological factors, such as sex hormones and diverse neurochemical pathways in the brain, as well as psychosocial and sociocultural factors [3]. Fluctuations in the concentrations of estradiol, progesterone, and their metabolites in the plasma and brain during the reproductive cycle in women increases the vulnerability to stressors and the development of anxiety and depression symptoms [4], as observed in the premenstrual period, postpartum, and during menopause [5]. Increases in the severity of physical, emotional, and psychological symptoms during the premenstrual period appear as part of premenstrual syndrome, as characterized by low concentrations of progesterone and estradiol [6] and alterations in neurotransmitters in the brain [7]. These changes trigger emotional disturbances, such as irritability, insomnia, anxiety, and depression, which impact the quality of life in women [8].

Preclinical studies have also identified sex differences in anxiety- and depression-like behaviors [9,10]. In females, anxiety- and depression-like behaviors are dependent on the phases of the estrus cycle [11,12]. Anxiety-like behavior increases during metestrus–diestrus, when estrogen concentrations are low, and it decreases during the proestrus–estrus phase, when the plasma concentrations of steroid hormones rise [13]. The metestrus–diestrus phase is considered equivalent to the premenstrual period [14] and resembles premenstrual syndrome in women [15]. Anxiety-like behavior throughout the estrus cycle in female mice and rats has led to research on substances that could be used as alternative therapies for treating the emotional symptoms that are characteristic of premenstrual syndrome [16].

The emotional and affective symptoms of premenstrual syndrome can be treated with anxiolytic and antidepressant drugs [17]. Serotonergic antidepressants are effective, including fluoxetine, citalopram, sertraline, and some tricyclic antidepressant drugs such as clomipramine [18]. However, a significant group of women do not respond to these treatments. In these women, the use of hormonal therapy with progestins and steroidal estrogens is an alternative treatment, but it is contraindicated for women with a risk of developing breast or endometrial cancer [6]. This is because of the oncogenic effects of the estradiol metabolites 2-hydroxy- and 16-hydroxy-estradiol [19,20] and significant side effects, such as an increased risk of cerebrovascular accidents, deep vein thrombosis, and venous thromboembolism [21], which limit the long-term use of this therapy. This highlights the importance of developing alternative therapies for the treatment of premenstrual syndrome that are derived from the secondary metabolites of plants such as genistein.

**Figure 1:**
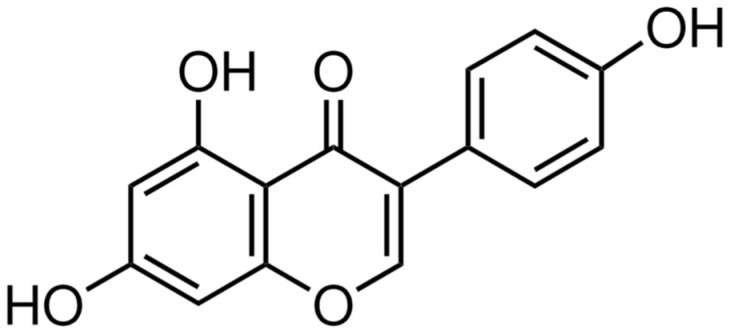
Chemical structure of the phytoestrogen genistein, which exerts several pharmacological effects, including antitumor effects; the regulation of glucose metabolism; a reduction in perimenopausal and postmenopausal hot flashes; antioxidant activity; protective effects against cardiovascular disease, diabetes, and osteoporosis; and the amelioration of menopausal symptoms such as anxiety and depression.

Diets rich in phytoestrogens are recommended to prevent the physiological and psychological symptoms present during the premenstrual period and menopause transition [22,23]. On this topic, preclinical research has proven that some natural compounds exert anxiolytic- and antidepressant-like effects in both male and female rats. The intake of dietary soy phytoestrogens produces anxiolytic-like effects in cycling female rats during the proestrus, but not the diestrus, phase [24]. These differences could be the result of evaluating dietary soy supplements rather than purified phytoestrogens, which prevents the control of the doses administered. The administration of purified phytoestrogens derived from soy such as genistein (Figure 1) produces anxiolytic-like effects in experimental animal models [25]. The administration of genistein (0.25, 0.5, or 1 mg/kg, i.p., for 4 days) reduces anxiety-like behavior in female rats subjected to a surgical menopause model [26], similarly to 17β-estradiol. In both cases, these effects were established through ERβ, since the antagonism of this receptor blocked the effect of genistein [27]. Furthermore, 0.25 mg/kg of genistein and daidzein, s.c. for 4 weeks, reduced anxiety-like behavior in ovariectomized rats without causing significant uterine tissue changes or the expression of uterine ERα and ERβ, while 17β-estradiol reduced anxiety-like behavior but increased the expression of both types of receptors [25]. It is important to highlight that the lack of effects of either genistein or daidzein on ERα expression suggests that they may be alternatives to 17β-estradiol for ameliorating anxiety-like behavior with a low risk of activating oncogenes or predisposing an individual to the development of certain types of cancer. Therefore, the study of phytoestrogens, particularly in females, will contribute to the development of therapies for ameliorating the emotional symptoms that occur during the premenstrual period.

Although the anxiolytic-like effects of genistein in ovariectomized rats have been previously reported, the effect of genistein on anxiety-like behavior during the metestrus–diestrus phase, which resembles the premenstrual period, as well as the impact on plasma concentrations of estradiol and progesterone, remains unexplored. In this sense, the present study contributes to the identification of the anxiolytic effect of genistein during the metestrus–diestrus phase, which could promote the development of anxiolytic drugs for women with premenstrual syndrome who are not candidates for hormonal therapy or do not respond to conventional anxiolytic drugs. In the present study, it was hypothesized that genistein may reduce anxiety-like behavior during the metestrus–diestrus phase, similarly to 17β-estradiol, but without causing significant changes in the plasma concentrations of estradiol or progesterone. The results contribute to the identification of the potential therapeutic activity of genistein for the treatment of premenstrual syndrome in women.

## 2. Materials and Methods

### 2.1. Ethics

All experimental methods followed the ethical recommendations of the Mexican norm NOM-062-ZOO-1999 [28] and the Guide for the Care and Use of Laboratory Animals of the National Research Council, USA, 2011 [29]. Additionally, the 3Rs (reduce, replace, and refine) of experimental research were considered to minimize animal discomfort [30] throughout the study. The general protocol obtained approval from the Internal Committee for the Care and Use of Laboratory Animals of the Institute of Health Sciences at Universidad Veracruzana, approval number: CICUAL-ICS-2023-02-02.

### 2.2. Animals

Fifty-six female Wistar rats, three months old and weighing 220–260 g, were included in this study. The rats were housed in Plexiglas cages (44 × 33 base and 20 cm high) and maintained in local housing facilities at 25 °C ± 1 °C under a 12 h/12 h light/dark cycle (lights on at 7:00 AM). Four to five rats were housed per cage. Purified water and standard rodent food (Agribrands Purina Co., Mexico City, Mexico) were supplied ad libitum. The guaranteed food analysis was as follows: 23.0% protein, 3.0% fat, 6.0% fiber, 47.4% NFE, 12.0% humidity, 7.0% ash, 1.0% calcium, and 0.6% phosphorus. The content was as follows: ground cereals, fish flour, cereal by-products, alfalfa, and cane molasses.

### 2.3. Vaginal Smears

To determine the regularity of ovarian cycles in the experimental subjects, vaginal smears were analyzed daily. Only rats with three consecutive regular cycles (4–5 days) were included in the experiments. The samples were obtained carefully by introducing the tip of a medicine dropper into the vagina, flushing saline in and out, and pouring the fluid onto microscope slides. The ovarian cycle phases (metestrus, diestrus, proestrus, and estrus) were determined using optical microscopy (40× magnification) to estimate the relative proportion of leukocytes, nucleated epithelial cells, and cornified epithelial cells.

### 2.4. Experimental Groups and Treatments

The rats with regular ovarian cycles were assigned to three independent groups. The first group, identified as the vehicle group (vehicle, *n* = 19), received corn oil (1 mL/kg, Mazola®, ACH Foods Mexico, Mexico City, Mexico), which was used to dissolve the genistein (Sigma-Aldrich, St. Louis, MO, USA) and 17β-estradiol (Sigma-Aldrich, St. Louis, MO, USA). The other two groups of rats received 0.09 mg/kg of genistein (genistein, *n* = 18) or 0.09 mg/kg of 17β-estradiol (17β-estradiol, *n* = 19). All treatments were injected subcutaneously in a single dose at a volume of 1 mL/kg. Sixty minutes after the corresponding injection, the rats were evaluated once using the elevated plus maze, open field, and light/dark box tests. The time that elapsed between the injection of the treatment and the behavioral tests and the doses of genistein and 17β-estradiol were selected from a dose–response study, in which a dose of 0.09 mg/kg was found to exert anxiolytic-like effects in Wistar rats after sixty minutes of subcutaneously administering the substances [26]. Additionally, the pharmacokinetic parameters showed that genistein has a high and rapid absorption and extensive distribution in the intestines; the half-life in the central and peripheral compartments is 2.1 min and 1.2 h with a bolus administration of 5 mg/kg [31], while after a single oral administration of 125 mg/kg of genistein, the half-life is 2.7 h [32]. These pharmacokinetic parameters support the 60 min pre-test interval, considering that genistein would have been absorbed, distributed, and still present in the body of the rats during the time in which the behavioral tests were performed.

Five minutes elapsed between each behavioral test. According to previous reports, after the last behavioral test, vaginal smears were obtained to confirm the ovarian cycle phase and then assign each rat to one of the following two subgroups: the proestrus–estrus phase, characterized by low anxiety and a high concentration of ovarian hormones, and the metestrus–diestrus phase, during which high anxiety and low concentrations of ovarian hormones are detected [33]. The latter phase is considered a physiological stage in rats that reproduces some of the same emotional and behavioral symptoms observed during premenstrual syndrome in women [15].

### 2.5. Behavioral Tests

One hour before the start of the behavioral tests, the cycling rats were maintained in the experimental room to acclimate them to the novel surroundings. All behavioral sessions were performed between 10:00 and 13:00 h. Each rat was evaluated once in a behavioral test battery, which sequentially included the elevated plus maze (EPM), locomotor activity test (LAT), and light/dark box (LDB). Digital video cameras (Sony DCR-SR42, 40× optical zoom, Carl Zeiss lens, San Diego, CA, USA) were installed above each apparatus to record the behavioral activity. Each test lasted 5 min, and approximately 4 min elapsed between tests. This protocol helped reduce the number of animals and has been validated to evaluate specific behavioral patterns, such as anxiety-like behavior and general motor activity in different apparatuses [34], which is in accordance with the ethical recommendations of the 3Rs [30].

At the end of each test session, the EPM, LAT, and LDB apparatuses were thoroughly cleaned with a 15% ethanol solution to eliminate any odors that could influence the behavior of the next rat.

Finally, two blind independent observers quantified the behavioral variables from video-recorded sessions until obtaining a 95% agreement. The quantification of the number of occurrences and the time spent in seconds in each of the selected variables was performed with an ex professo software version 1.0.

#### 2.5.1. Elevated Plus Maze

The EPM was constructed of wood and sealed with acrylic paint. It had two white, open (50 cm length × 10 cm width) and two black, closed (50 cm length × 10 cm width × 40 cm height) arms placed 50 cm above the floor. On the day of the test, each rat was placed in the center of the EPM, facing an open arm. The evaluated variables were (a) the time spent in the open arms, (b) the number of entries into the open arms, (c) the number of entries into the closed arms, (d) the total number of entries into the arms (open + closed arms), and (e) the anxiety index, as follows: AI = 1 − [(time spent on the open arms/300 s) + (entries into the open arms/total number of entries)/2]. The results section reports only the time spent in the open arms and the AI because these two variables are the principal robust predictors of an anxiolysis state [35], and the other variables are included in the AI.

#### 2.5.2. Locomotor Activity Test

Each rat was gently placed into a box with a 44 cm × 33 cm base and a 20 cm height. The box floor was delineated into twelve squares (11 × 11 cm) to evaluate crossing, grooming, and rearing. The evaluated variables were (a) crossings, or the number of times that the rat moved from one square to another with its hind legs; (b) rearing, or the time spent in a vertical posture relative to the cage floor; and (c) grooming, or the time spent in nose/face grooming, head washing, paw licking, leg licking, body grooming/scratching, or tail/genital grooming.

#### 2.5.3. Light/Dark Box

The LDB was constructed of glass (80 cm × 40 cm base and 40 cm high walls). The box had two equal chambers (40 cm × 40 cm × 40 cm) connected by a doorway (10 cm × 10 cm) that allowed the rats to freely cross into both chambers. The walls of the light compartment were covered with a white film, and it was illuminated (40 W white light). The walls of the dark compartment were covered with a black film, and it was not illuminated.

Each rat was placed in the middle of the lighted compartment facing the doorway. The evaluated variables were (a) the latency to the first entry into the dark compartment; (b) the total time spent in the light compartment; and (c) the total number of entries into the light compartment. These variables were scored with the assumption that they provide a reliable measure of anxiety-like behavior.

### 2.6. Determination of Plasma Concentration of Estradiol and Progesterone

Ten minutes after the last behavioral test, each rat was deeply anesthetized with sodium pentobarbital (60 mg/kg, i.p., Cheminova of Mexico, Mexico City, Mexico; Reg. SAGARPA Q-7048-044), and blood samples were obtained through an intracardial punction using a 5 mL syringe with a 22G needle. The samples were centrifuged at 3500 rpm/5 min to obtain serum, which was then stored at −20 °C until further analyses. The estradiol and progesterone concentrations were obtained using the ELISA method by following the manufacturer’s protocols for the estradiol and progesterone kits (Alpco Diagnostic^®^, Salem, NH, USA). The intra- and inter-assay variations were less than 5% for both hormones. The sensitivity of the kits used to measure the estradiol concentration ranged from 10 pg/mL to 3200 pg/mL, and that of the kits used to measure progesterone ranged from 0.3 ng/mL to 60 ng/mL.

### 2.7. Statistical Analysis

The assumptions of the normality and homogeneity of the data were verified, and then the data were analyzed using a two-way analysis of variance (two-way ANOVA) for independent groups, with the treatment and ovarian cycle phase as the factors. Values of *p* ≤ 0.05 in the ANOVA were followed by the Student–Newman–Keuls post hoc test. The results are presented as the mean ± standard error of the mean. The statistical analysis was performed using SigmaPlot, version 12.0^®^ (Systat Software, Chicago, IL, USA).

## 3. Results

### 3.1. Grouping of Ovarian Cycle Phases According to Treatments

The analysis of vaginal smears allowed the ovarian cycle phase to be identified for each subgroup (i.e., proestrus–estrus and metestrus–diestrus), as indicated in Table 1.

### 3.2. Effect of Treatments on Anxiety-like Behavior in the EPM

**Figure 2:**
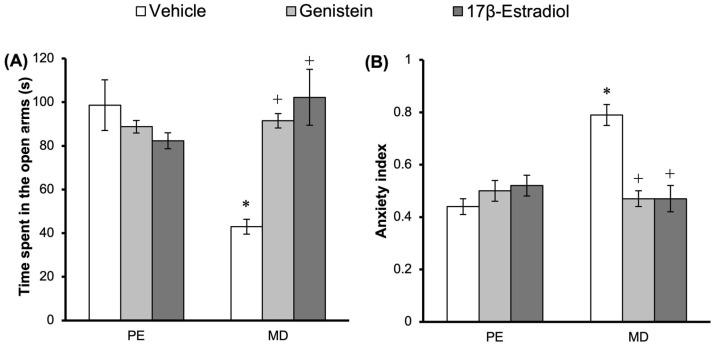
Elevated plus maze: treatment with genistein or 17β-estradiol increased the time spent in the open arms (**A**) and reduced the anxiety index (**B**) compared to the vehicle group during the MD phase. * *p* < 0.05 vs. vehicle—PE; + *p* < 0.05 vs. vehicle—MD. Student–Newman–Keuls post hoc test. PE, proestrus–estrus phase; MD, metestrus–diestrus phase.

The statistical analysis of the time spent in the open arms of the EPM did not show significant differences according to the ovarian cycle phase factor (*F*_1,50_ = 3.378; *p* = 0.072). However, significant differences were detected according to the treatment factor (*F*_2,50_ = 5.199; *p* = 0.009) and the interaction among factors (*F*_2,50_ = 14.584; *p* = 0.001). The post hoc test revealed that vehicle-treated rats in the metestrus–diestrus phase spent less time in the open arms compared with vehicle-treated rats in the proestrus–estrus phase. This effect (detected in metestrus–diestrus rats) was prevented in the rats treated with genistein or 17β-estradiol, which maintained similar values to those detected in proestrus–estrus rats. Genistein and 17β-estradiol produced no significant changes in this variable in proestrus–estrus rats (Figure 2A).

In support, the analysis of the anxiety index revealed significant differences according to the ovarian cycle phase factor (*F*_1,50_ = 8.221; *p* = 0.006), treatment factor (*F*_2,50_ = 6.955; *p* = 0.002), and interaction among factors (*F*_2,50_ = 16.942; *p* = 0.001). The post hoc test revealed that vehicle-treated rats in the metestrus–diestrus phase had a higher anxiety index than vehicle-treated rats in the proestrus–estrus phase. This effect (identified in metestrus–diestrus rats) was prevented in the rats treated with genistein or 17β-estradiol, which reached similar values to those observed in proestrus–estrus rats. The proestrus–estrus rats treated with genistein or 17β-estradiol did not show significant changes in this variable compared with the vehicle group (Figure 2B).

### 3.3. Effect of Treatments on Crossing, Rearing, and Grooming in the LAT

Table 2 shows the number of crossings and the time spent rearing and grooming in the LAT. The analysis of crossings revealed no significant changes according to the ovarian cycle phase factor (*F*_1,50_ = 0.109; *p* = 0.742), treatment factor (*F*_2,50_ = 0.741; *p* = 0.482), or interaction among factors (*F*_2,50_ = 0.589; *p* = 0.559).

The time spent rearing revealed no significant differences according to the ovarian cycle phase factor (*F*_1,50_ = 0.124; *p* = 0.726), nor the interaction among factors (*F*_2,50_ = 0.163; *p* = 0.850). However, significant differences according to the treatment factor were observed (*F*_2,50_ = 4.078; *p* = 0.023). The 17β-estradiol-treated rats spent less time rearing compared with the vehicle- and genistein-treated rats.

Regarding the analysis of the time spent grooming, significant differences were revealed according to the ovarian cycle phase factor (*F*_1,50_ = 55.623; *p* = 0.001), treatment factor (*F*_2,50_ = 10.549; *p* = 0.001), and interaction among factors (*F*_2,50_ = 5.755; *p* = 0.006). The vehicle-treated rats in the metestrus–diestrus phase spent less time grooming compared with vehicle-treated rats in the proestrus–estrus phase. Interestingly, treatment with genistein or 17β-estradiol attenuated the reduced time spent grooming observed in the metestrus–diestrus phase, which was significantly different compared with the vehicle-treated rats in this same ovarian cycle phase. In contrast, the proestrus–estrus rats treated with genistein or 17β-estradiol did not show significant changes in this variable compared with the vehicle group.

### 3.4. Effect of Treatments on Anxiety-like Behavior in the LDB

**Figure 3:**
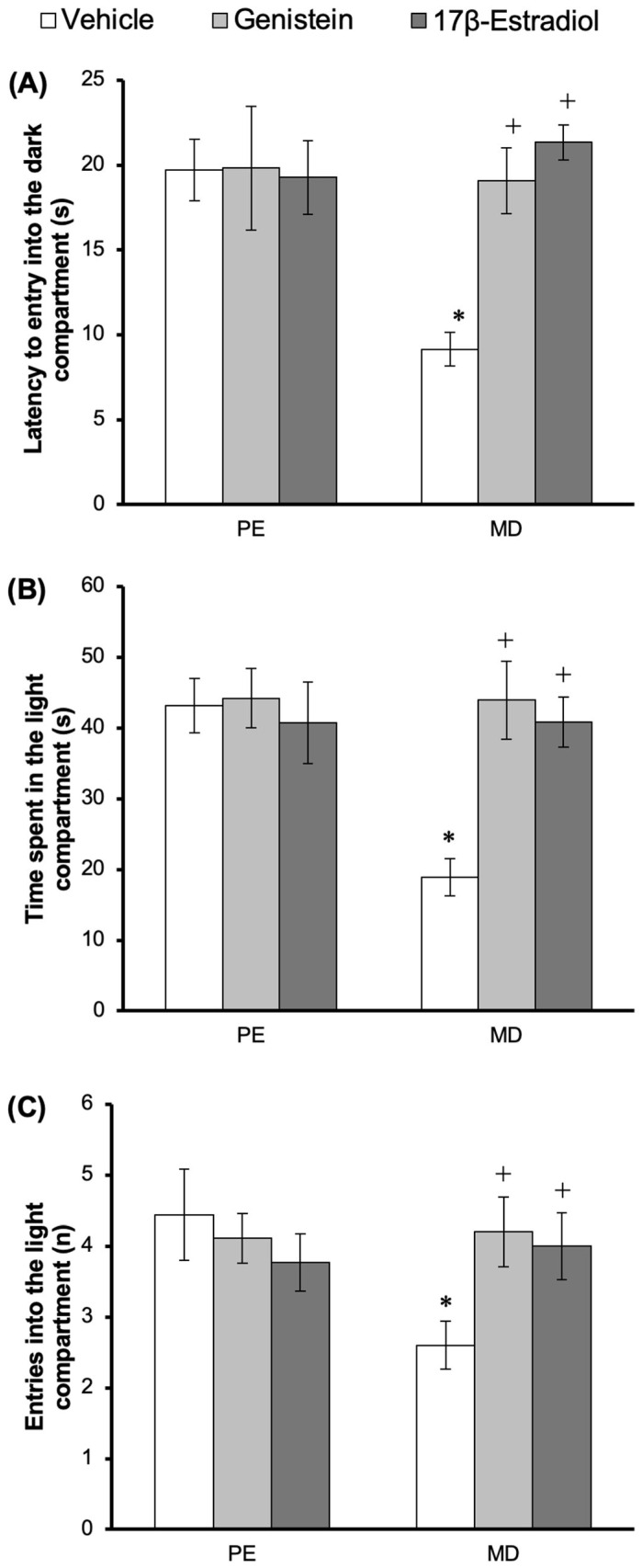
Light/dark test: treatment with genistein or 17β-estradiol increased the latency to entry into the dark compartment (**A**) and the time spent in (**B**) and number of entries into (**C**) the light compartment compared to the vehicle group in the MD phase. * *p* < 0.05 vs. vehicle—PE; + *p* < 0.05 vs. vehicle—MD. Student–Newman–Keuls post hoc test. PE, proestrus–estrus phase; MD, metestrus–diestrus phase.

The statistical analysis of the latency to entry into the dark compartment revealed significant differences according to the cycle phase factor (*F*_1,50_ = 10.907, *p* < 0.002), treatment factor (*F*_2,50_ = 15.549, *p* = 0.002), and interaction among factors (*F*_2,50_ = 16.819, *p* < 0.001). The post hoc test revealed that vehicle-treated rats in the metestrus–diestrus phase displayed a shorter latency for entering the dark compartment compared with vehicle-treated rats in the proestrus–estrus phase. This effect (detected in metestrus–diestrus rats) was prevented in rats treated with genistein or 17β-estradiol, which reached similar values to those observed in proestrus–estrus rats. No significant changes in this variable were detected in the proestrus–estrus rats treated with genistein or 17β-estradiol (Figure 3A).

Regarding the number of entries into the light compartment, the analysis revealed no significant differences according to the cycle phase factor (*F*_1,50_ = 2.207, *p* = 0.144) or treatment factor (*F*_2,50_ = 1.158, *p* = 0.322). However, significant differences were observed according to the interaction among factors (*F*_1,50_ = 3.794, *p* = 0.029). The post hoc test revealed that vehicle-treated rats in the metestrus–diestrus phase had a lower value for this variable compared with vehicle-treated rats in the proestrus–estrus phase. This effect (observed in metestrus–diestrus rats) was prevented by the genistein and 17β-estradiol treatments, as the rats that received these treatments reached similar values to those observed in proestrus–estrus rats. No significant differences in this variable were detected in proestrus–estrus rats treated with genistein or 17β-estradiol (Figure 3B).

Finally, changes in the total time spent in the light compartment were observed according to the cycle phase factor (*F*_1,50_ = 20.210, *p* < 0.001), treatment factor (*F*_2,50_ = 19.0571, *p* = 0.001), and interaction among factors (*F*_1,50_ = 19.921, *p* < 0.001). The post hoc test revealed that vehicle-treated rats in the metestrus–diestrus phase spent less time in the light compartment compared with vehicle-treated rats in the proestrus–estrus phase. Interestingly, this effect was prevented by the genistein and 17β-estradiol treatments. No significant effect of the treatments on this variable was detected in proestrus–estrus rats (Figure 3C).

### 3.5. Effect of the Treatments on Estradiol and Progestogen Plasma Concentrations

Table 3 shows the plasma concentrations of estradiol and progesterone according to the ovarian cycle phases and treatments. The statistical analysis of the estradiol concentration revealed significant differences according to the cycle phase factor (*F*_1,50_ = 10.484, *p* = 0.002), treatment factor (*F*_2,50_ = 417.262, *p* < 0.001), and interaction among factors (*F*_2,50_ = 23.113, *p* < 0.001). The post hoc test showed that the vehicle- and genistein-treated rats in the metestrus–diestrus phase had a lower estradiol concentration than the respective group in the proestrus–estrus phase. Interestingly, genistein did not modify the estradiol concentration in the proestrus–estrus or metestrus–diestrus phases compared with the vehicle group in the respective phases. However, the 17β-estradiol treatment significantly increased the estradiol concentration in both phases of the ovarian cycle compared with the respective vehicle and genistein groups in the same phase.

With respect to the plasma concentration of progesterone, the statistical analysis did not reveal significant differences according to the cycle phase factor (*F*_1,50_ = 1.594, *p* = 0.213), treatment factor (*F*_2,50_ = 2.418, *p* = 0.099), or interaction among factors (*F*_2,50_ = 0.021, *p* = 0.979).

## 4. Discussion

The present study evaluated the effects of the phytoestrogen genistein on anxiety-like behavior and the plasma concentrations of estradiol and progesterone while considering the ovarian cycle phase. A dose of 0.09 mg/kg of genistein reduced the anxiety-like behavior characteristic of the metestrus–diestrus phase according to the EPM and LDB tests, without causing significant changes in spontaneous motor activity in the LAT. These effects produced by genistein were similar to those produced by 17β-estradiol. No significant changes were detected in the anxiety-like behavior associated with genistein or 17β-estradiol during the proestrus–estrus phase. As expected, genistein did not increase the plasma concentrations of estradiol or progesterone, while the administration of 17β-estradiol produced an increase in the plasma concentration of estradiol without changing the progesterone concentration. These results show the intrinsic estrogenic effect of genistein associated with anxiolytic-like effects in the metestrus–diestrus phase, suggesting its potential use for ameliorating anxiety symptoms during premenstrual syndrome in women. It is true that the anxiolytic-like effect of genistein has been previously reported in female and male subjects under different experimental conditions; however, the present study adds information about the anxiolytic effects of genistein in a specific physiological state of females, characterized by low concentrations of ovarian hormones and a high vulnerability to stress and the development of emotional disorders. This shows that genistein could be used in the design of natural pharmacological therapies focused on the treatment of emotional alterations that occur during premenstrual dysphoric syndrome in women.

Throughout the ovarian cycle, physiological and hormonal changes may negatively impact emotional states [36]. During the premenstrual period, women are more vulnerable to vasomotor symptoms, insomnia, depression, and anxiety, which are associated with low concentrations of steroid hormones such as estradiol [37]. In this way, hormonal therapy can be used to ameliorate such changes that naturally occur before menstruation; however, estrogen therapy may be associated with significant side effects, including endometrial and breast cancer [38,39], which limits the use of this therapy in subjects with a familial history of estrogen-dependent cancer. Alternatively, anxiolytic and antidepressant drugs have also been used to ameliorate the emotional symptoms associated with the ovarian cycle; however, a significant group of females develop side effects that limit the use of these drugs [40].

At the preclinical level, rats in the metestrus–diestrus phase are considered to be in a physiological condition that resembles premenstrual syndrome in women; in this phase in rats, the hormone levels decrease. Indeed, in rats as well as in women, this period is reportedly associated with a greater vulnerability to anxiety, hopelessness, and aggressive behaviors [41]. Contrarily, preclinical and clinical studies have identified that phytoestrogens contained in plants such as soja produce similar physiological and behavioral effects as estradiol, supporting the hypothesis that these substances could be used as an alternative to steroidal estrogens, anxiolytics, and antidepressant drugs for the treatment of emotional symptoms that occur during the premenstrual period.

The results of the present study expand the notion that genistein has a potential anxiolytic-like effect similar to 17β-estradiol when low concentrations of ovarian hormones are detected, as occurs during the metestrus–diestrus phase in rats, which is equivalent to the premenstrual period in women [15]. Genistein injected into ovariectomized rats subcutaneously at a similar dose to that of 17β-estradiol (0.09 mg/kg) increased the time spent in the open arms and reduced the AI in the EPM, indicating an anxiolytic-like effect [27]. Similar effects have been reported when anxiolytic drugs such as diazepam, olanzapine, risperidone, and natural metabolites from plants such as flavonoids have been evaluated using the EPM [42,43]. Consistently, other studies have demonstrated the anxiolytic-like effect of genistein under diverse experimental conditions. For example, in adult male rats with a traumatic brain injury, 5, 10, and 20 mg/kg of genistein, administered i.p. once a day for 14 days, decreased anxiety-like behaviors and reduced the apoptosis and brain inflammation induced by the traumatic brain injury [44]. In addition, 4 and 8 mg/kg of genistein administered i.p. for 7 days reduced anxiety in the EPM in adult male rats with post-traumatic stress disorder produced by electric shock stress, possibly due to an increase in serotonergic transmission in the amygdala [45]. These data show that genistein produces anxiolytic-like effects when administered in a wide range of dosages; these effects may depend on the sex, route of administration, and duration of treatment, among others.

In support, genistein and 17β-estradiol significantly lengthened the latency of entering the dark compartment and increased the time that the rats remained in the light compartment during the metestrus–diestrus phase in the LDB in this study. The light compartment is considered an aversive stimulus that increase anxiety in vulnerable subjects [46]. Therefore, a longer latency for entering the dark compartment and a greater time spent in the light area are considered anxiolytic-like effects [46]. In this sense, animals treated with anxiolytic drugs such as benzodiazepines and selective serotonin reuptake inhibitors demonstrated the same behaviors in the LDB [47] to those treated with genistein or 17β-estradiol in the present study. Therefore, the behavioral effects detected in the metestrus–diestrus phase support the anxiolytic-like effect of genistein and 17β-estradiol measured in two experimental models of anxiety.

Spontaneous motor activity was also evaluated to rule out or identify motor effects such as hypoactivity or hyperactivity, which may have interfered with exploration in the EPM and LDB. The absence of changes in the number of crossings allowed us to rule out any motor influence in the anxiety tests. Therefore, the results identified in both anxiety tests can be attributed to effects on the emotional status of the animals, confirming the anxiolytic-like effect of genistein and 17β-estradiol. Additionally, grooming behavior is low in rats with high levels of anxiety-like behavior, as it occurred in the vehicle-treated rats during the metestrus–diestrus phase, where low concentrations of estrogens were reported. Genistein and 17β-estradiol restored the grooming behavior to similar values as those identified in the vehicle-treated rats in the proestrus–estrus phase. These behavioral effects of the treatments on grooming indicate an anxiolytic-like effect [26], as has been reported when anxiolytic drugs (e.g., diazepam, clozapine, and risperidone) are administered to experimental subjects [48] and evaluated in behavioral tests. Thus, substances with anxiolytic activity increase the grooming behavior, as was seen in the rats treated with genistein or 17β-estradiol in the present study. Rearing is a behavior indicative of exploration, which may or may not be modified by anxiolytic drugs. In long-term ovariectomized rats, the treatments with genistein or 17β-estradiol significantly increased the rearing behavior; however, treatment with other anxiolytic drugs (e.g., diazepam, chrysin, or the neurosteroid allopregnanolone) had no effect on the rearing behavior [16]. This is in concordance with results of the present study and indicates that the modification of rearing by anxiolytic drugs could be dependent on the physiological condition of the experimental subjects at the time of evaluation.

Interestingly, diverse reports suggest that genistein can activate both estrogen receptor isoforms (i.e., ERα and ERβ), although genistein binds to ERβ with a 20-fold higher affinity compared with its affinity for ERα [49]. This suggests that genistein could pose a low risk of activating oncogenes for the development of endometrial and breast cancers that are dependent on ERα activation. For example, genistein suppresses tumor growth in endometrial cancer and significantly reduces the expression of ERα in tumors [50]. Genistein may be more beneficial versus estrogen therapy for ameliorating anxiety symptoms with low side effects because of its minimal interactions with ERα, which does not occur with 17β-estradiol [51]. It has been reported that the symptoms of premenstrual syndrome appear when progesterone levels predominate over estrogen levels [52]. In this study, it is necessary to highlight that none of the treatments produced significant changes in the plasma concentrations of progesterone, indicating that genistein and estradiol exhibited no biological activity in modulating the progesterone concentration. However, it is important to mention that progesterone is used as a treatment for endometrial cancer, and its effect is mediated by binding to its receptors, while genistein exerts an antitumoral effect, possibly by increasing the expression of progesterone receptors [50]. Clinical studies have reported that low doses of phytoestrogens (oral consumption of ~25 mg of isoflavones per day) in women do not affect cell proliferation [53], and these studies have not reported any significant side effects [54]. It has even been reported that some phytoestrogens, including genistein, are safe for breast cancer patients when administered over a long time [55] and that they produce promising beneficial effects for cancer treatment [56]. These findings support the hypothesis that genistein could contribute to the development of natural therapies for the treatment of anxiety symptoms during the premenstrual period in women, with a low risk of causing significant side effects.

On the other hand, it is known that standard rat food includes soy and alfalfa, which contain phytoestrogens that may modify physiological processes, increase the spine synapse density in the CA1 area of the hippocampus [57], and reduce anxiety-like behavior. Even though the physiological and behavioral effects produced by dietary phytoestrogens were not evaluated in this study, it was still possible to detect anxiolytic-like effects in the rats treated with genistein and 17*β*-estradiol [27]. The possible effect of dietary phytoestrogens on anxiety was disregarded, considering that significant differences in anxiety-like behavior were detected between the genistein- and 17*β*-estradiol-treated groups. Furthermore, the vehicle-treated group was exposed to the dietary phytoestrogens. Therefore, it was considered that, under our experimental conditions, the presence of dietary phytoestrogens was not sufficient to interfere with the detection of anxiolytic-like effects caused by the investigated treatments in cycling rats. However, future studies could be performed to rule out or identify the contribution of dietary phytoestrogens to the anxiolytic-like effects in cycling rats treated with genistein or 17*β*-estradiol injections.

It is important to mention that there are some limitations in the present study, as follows: (a) The ovarian cycle phases were confirmed using vaginal smears after behavioral tests, which could have produced a certain level of stress and affected the cycle phases and hormone levels; however, it was not possible to eliminate this factor because it was inherent to the experimental manipulations. Although we cannot disregard the possible influence of this manipulation on the cycle phase and hormone concentrations, all the experimental groups (including the vehicle group) were subjected to the same manipulations throughout the study and we still detected the differences in plasma concentrations and pharmacological responses according to the ovarian cycle phase, as has been previously reported [34,58,59]. It is also important to highlight that, before the experimental manipulations, the ovarian cycle phase was monitored for three consecutive regular cycles, enabling the ovarian cycle phase in which the rat would perform the behavioral tests to be predicted, and this was confirmed with the vaginal smear at the end of the behavioral tests. This is supported by the low variation in the number of animals per group, as shown in Table 1; however, this limitation could have exerted some influence on the stress induced by the behavioral tests on the ovarian cycle phase and the plasma concentrations of ovarian hormones. (b) The mechanism of action underlying the anxiolytic-like effect of genistein was not evaluated; however, it is suggested that the anxiolytic-like effects of genistein could be related to the activation of central ERβ, considering that ERα is more involved in the regulation of peripheral processes [49]. In addition, it has been reported that the anxiolytic-like effects of genistein in ovariectomized rats are blocked by the antagonism of ERβ [60]. Nevertheless, considering the rapid effects of genistein and 17*β*-estradiol in reducing anxiety-like behavior, it is not possible to disregard the participation of the GPER, which has a similar affinity for both substances [61]. In this way, the neuronal processes that implicate the activation of cellular membranes could induce a rapid reaction, and this could modulate the emotional state in a short time through the activation of the GPER, which could have been involved in the observed results of the present study. This hypothesis requires testing through additional specific protocols. (c) Finally, an additional limitation of the present study is the use of single doses and the administration of treatments through the subcutaneous route. There are preclinical and clinical reports showing that genistein has a wide range of dosages that can produces anxiolytic-like effects, which may depend on the sex, route of administration, and duration of treatment. However, the present study adds information about the rapid effects of a single injection of genistein, indicating that this substance could have therapeutic applications for the treatment of anxiety symptoms that occur acutely during the premenstrual period in women. However, this needs to be more thoroughly studied before genistein can be recommended for clinical use in humans.

## 5. Conclusions

The phytoestrogen genistein exerted anxiolytic-like effects during the metestrus–diestrus phase of rats, an ovarian cycle phase that resembles the premenstrual period in women. Genistein could be considered for the development of an anxiolytic drug to treat emotional symptoms during the premenstrual period in women, with a reduced possibility of producing significant estrogen-dependent side effects in comparison with the steroidal estrogens used in hormone replacement therapy. However, more data are needed to check for the innocuity of oral or subcutaneous genistein treatment in women with pre-existing breast cancer cells, and to perform medical supervision when phytoestrogens are used for clinical purposes. Only then would it be possible to consider genistein as a safe and effective alternative for the treatment of anxiety symptoms, as well as for overcoming the current barriers to its recommendation for clinical use.

## Figures and Tables

**Table 1:** Number of rats per subgroup and group according to the ovarian cycle phase.

Group	Subgroups		
	Proestrus–Estrus	Metestrus–Diestrus	Total
Vehicle	9	10	19
Genistein, 0.09 mg/kg	9	9	18
17β-Estradiol, 0.09 mg/kg	9	10	19

**Table 2:** Crossing, rearing, and grooming of rats according to the ovarian cycle phase and treatment in the locomotor activity test.

Variable/Group	Subgroups	
	**Proestrus–Estrus**	**Metestrus–Diestrus**
**Crossing**		
Vehicle	46.00 ± 4.96	43.20 ± 2.52
Genistein, 0.09 mg/kg	47.44 ± 2.40	44.80 ± 1.80
17β-Estradiol, 0.09 mg/kg	46.78 ± 3.25	49.78 ± 2.44
**Rearing**		
Vehicle	21.92 ± 2.66	21.53 ± 2.85
Genistein, 0.09 mg/kg	23.52 ± 2.40	23.73 ± 2.80
17β-Estradiol, 0.09 mg/kg	15.46 ± 1.83 ^+^	17.82 ± 2.23 ^+^
**Grooming**		
Vehicle	24.57 ± 1.29	9.51 ± 0.70 *
Genistein, 0.09 mg/kg	26.22 ± 2.82	21.45 ± 1.63 ^#^
17β-Estradiol, 0.09 mg/kg	26.36 ± 1.69	17.87 ± 3.31 ^#^

^+^ *p* < 0.05 vs. vehicle and genistein groups; * *p* < 0.05 vs. proestrus–estrus phase in the same group; ^#^ *p* < 0.05 vs. vehicle group in the same phase. Data are expressed as the mean ± standard error of the mean. Two-way ANOVA, post hoc Student–Neuman–Keuls test.

**Table 3:** Plasma concentration of estradiol and progesterone according to the treatments and ovarian cycle phase.

Hormone/Group	Subgroups	
	**Proestrus–Estrus**	**Metestrus–Diestrus**
**Estradiol, pg/mL**		
Vehicle	26.36 ± 1.08	11.06 ± 0.63 *
Genistein, 0.09 mg/kg	29.46 ± 1.32	13.89 ± 1.81 ^+^
17β-Estradiol, 0.09 mg/kg	75.68 ± 4.64 ^#^	88.64 ± 7.49 ^#^
**Progesterone, ng/mL**		
Vehicle	31.29 ± 2.35	29.52 ± 2.64
Genistein, 0.09 mg/kg	33.83 ±1.72	31.83 ± 1.76
17β-Estradiol, 0.09 mg/kg	35.25 ± 2.16	32.95 ± 1.83

* *p* < 0.05 vs. proestrus–estrus phase in the same group; ^+^ *p* < 0.05 vs. proestrus–estrus phase in the same group; ^#^ *p* < 0.05 vs. vehicle and genistein groups in the same phase. Data are expressed as the mean ± standard error of the mean. Two-way ANOVA, post hoc Student–Neuman–Keuls test.

## Data Availability

The data will be made available by the corresponding authors upon request. The data are not publicly available due to the requirement of the project privacy before the close of the general project.

## Abbreviations

The following abbreviations are used in this manuscript:

ERα	estrogen receptor α
ERβ	estrogen receptor β
EPM	elevated plus maze
LAT	locomotor activity test
LDB	light/dark box
AI	anxiety index
GPER	G-protein-coupled estrogen receptor
i.p.	intraperitoneal injection
s.c.	subcutaneous injection
